# Gene expression network analyses during infection with virulent and avirulent *Trypanosoma cruzi* strains unveil a role for fibroblasts in neutrophil recruitment and activation

**DOI:** 10.1371/journal.ppat.1008781

**Published:** 2020-08-18

**Authors:** Antonio Edson R. Oliveira, Milton C. A. Pereira, Ashton T. Belew, Ludmila R. P. Ferreira, Larissa M. N. Pereira, Eula G. A. Neves, Maria do Carmo P. Nunes, Barbara A. Burleigh, Walderez O. Dutra, Najib M. El-Sayed, Ricardo T. Gazzinelli, Santuza M. R. Teixeira

**Affiliations:** 1 Departamento de Bioquímica e Imunologia, Universidade Federal de Minas Gerais, Belo Horizonte, MG, Brazil; 2 Centro de Pesquisas Rene Rachou, Fundação Oswaldo Cruz, Belo Horizonte, MG, Brazil; 3 Department of Cell Biology and Molecular Genetics and Center for Bioinformatics and Computational Biology, University of Maryland, College Park, Maryland, United States of America; 4 Departamento de Morfologia, Universidade Federal de Minas Gerais, Belo Horizonte, MG, Brazil; 5 Departamento de Clínica Médica, Faculdade de Medicina, Universidade Federal de Minas Gerais, Belo Horizonte, MG, Brazil; 6 Department of Immunology and Infectious Diseases, Harvard T. H. Chan School of Public Health, Boston, Massachusetts, United States of America; University of California, Los Angeles, UNITED STATES

## Abstract

Chagas disease is caused by *Trypanosoma cruzi*, a protozoan parasite that has a heterogeneous population composed of a pool of strains with distinct characteristics, including variable levels of virulence. In previous work, transcriptome analyses of parasite genes after infection of human foreskin fibroblasts (HFF) with virulent (CL Brener) and non-virulent (CL-14) clones derived from the CL strain, revealed a reduced expression of genes encoding parasite surface proteins in CL-14 compared to CL Brener during the final steps of the intracellular differentiation from amastigotes to trypomastigotes. Here we analyzed changes in the expression of host genes during *in vitro* infection of HFF cells with the CL Brener and CL-14 strains by analyzing total RNA extracted from cells at 60 and 96 hours post-infection (hpi) with each strain, as well as from uninfected cells. Similar transcriptome profiles were observed at 60 hpi with both strains compared to uninfected samples. However, at 96 hpi, significant differences in the number and expression levels of several genes, particularly those involved with immune response and cytoskeleton organization, were observed. Further analyses confirmed the difference in the chemokine/cytokine signaling involved with the recruitment and activation of immune cells such as neutrophils upon *T*. *cruzi* infection. These findings suggest that infection with the virulent CL Brener strain induces a more robust inflammatory response when compared with the non-virulent CL-14 strain. Importantly, the RNA-Seq data also exposed an unexplored role of fibroblasts as sentinel cells that may act by recruiting neutrophils to the initial site of infection. This role for fibroblasts in the regulation of the inflammatory response during infection by *T*. *cruzi* was corroborated by measurements of levels of different chemokines/cytokines during *in vitro* infection and in plasma from Chagas disease patients as well as by neutrophil activation and migration assays.

## Author summary

*Trypanosoma cruzi* is the causative agent of Chagas disease, a debilitating and often life-threatening illness that affects 6 to 7 million people mainly in Latin America. The parasite, transmitted to humans by an insect vector, needs to invade different cells from the infected person in order to multiply and spread the infection to various organs, including the heart and the gut. In this study, we investigated how the host cell responds to the infection by analyzing changes in the expression of human genes in fibroblasts infected with the CL Brener and CL-14 strains, which are strains that present highly distinct virulent phenotypes during infection in mice. We showed that human fibroblasts build a strong immune response upon infection by *T*. *cruzi* and that this response is different depending on the parasite strain: infection with the virulent CL Brener strain induces a more robust inflammatory response compared with the infection with the avirulent CL-14 strain. We also showed that, in response to the infection, fibroblasts produce molecules that can recruit and activate neutrophils, which are important immune cells that controls the infection.

## Introduction

*Trypanosoma cruzi* is the etiological agent of Chagas disease, a debilitating and often life-threatening disease that affects 6 to 7 million people (http://www.who.int/en/news-room/fact-sheets/detail/chagas-disease). Due to increasing migration and the lack of efficient treatment of chronically infected individuals, Chagas disease has spread outside endemic countries in Latin America and reached southern areas of the United States as well as other countries like Australia, Canada and Spain. The *T*. *cruzi* population displays high genetic diversity with strains exhibiting distinct biological characteristics such as morphology, growth rate, virulence, tissue tropism, drug sensitivity and antigenic profile [1]. Most strains and clones can be classified into six major clades, named discrete typing units (DTUs I to VI) [2] or TcI–TcVI [3]. Genome sequencing of the clone CL Brener, a TcVI strain, showed it is a hybrid strain containing two genotypes that belong to TcII and TcIII [4]. CL-14 is another clone derived from the CL strain that also belongs to the TcVI group, but in contrast to CL Brener, presents low virulence and is non-pathogenic, even when inoculated into newborn [5–7] or immune deficient, CD8^-/-^ mice [8]. Importantly, after inoculation in adult animals, CL-14 induces protective immunity and prevents the development of parasitemia and mortality following challenge with virulent *T*. *cruzi* strains [6,9,10].

We have previously used comparative transcriptome profiling of parasite genes to investigate the molecular basis of the difference between a virulent (CL Brener) and an avirulent (CL-14) *T*. *cruzi* strain. The study showed that, in contrast to the outcomes of the infection *in vivo*, CL Brener and CL-14 trypomastigotes are able to infect human fibroblasts *in vitro* with similar efficiencies [11]. At later time points of the infection, however, differentiation of the avirulent CL-14 amastigotes to extracellular trypomastigotes occurs at much lower pace, resulting in reduced number of trypomastigotes released by the CL-14 infected cells compared to the CL Brener infection. Accordingly, only few differences in the expression profiling of parasite genes were observed between the two parasite strains in the first 60 hpi. In contrast, at 96 hpi, when amastigotes from CL Brener are readily differentiating into motile trypomastigotes, the delayed differentiation of CL-14 is reflected by major differences in the transcriptome of both strains, particularly regarding the expression of genes encoding surface protein families such as trans-sialidases (TS), mucins and the mucin associated surface proteins (MASPs). These large gene families, some of them present with more than 1,000 copies in the genome [4], have been investigated by several groups. TS, MASP and mucins are essential components of the trypomastigote surface involved with the establishment of the infection in the mammalian host as well with escape mechanisms from the host immune response [12]. Thus, our previous comparative transcriptome study indicated that the avirulent phenotype of CL-14 may be due, at least in part, to inhibited expression of CL-14 virulence genes, most of them encoding surface proteins whose expression is associated with the transition of amastigotes to trypomastigotes [11].

Throughout the infection process, i.e., following attachment of motile trypomastigotes to the host cell surface, penetration, escape from the parasitophorous vacuole and subsequent replication of amastigotes in the cytoplasm, the host responds by activating intracellular pathways to control the infection [13]. Production of proinflammatory cytokines constitutes an essential event required for the development of an efficient innate and adaptive immune responses [14]. Several authors have described induced expression of type I interferon (IFN) genes as an early response during *in vitro* infection of human fibroblasts by different *T*. *cruzi* strains [15] as well as in bone marrow-derived macrophages (BMMs) [16]. Besides immune response genes, transcriptome studies based on microarray data [15–26] and RNA-Seq analyses [27,28] also identified interconnected metabolic networks centered around host energy production, nucleotide metabolism, pteridine biosynthesis and fatty acid oxidation as key cellular processes that are modified to fuel intracellular *T*. *cruzi* growth [29]. Importantly, a genome-wide functional RNAi screen further confirmed the capacity of the parasite to manipulate host metabolic and signalling functions and identified the host kinase Akt as a critical signaling molecule required for parasite replication [30].

Here, we compared the transcriptome profiling and interaction networks based on coding and non-coding genes from the nonphagocytic human foreskin fibroblast (HFF) infected with CL Brener and CL-14. Together with previously published analyses derived from the parasite RNA-Seq data, our results revealed further differences in the complex host-parasite equilibrium that is established during infection with distinct *T*. *cruzi* strains, particularly at late infection time points. Importantly, our comparative transcriptome analyses also unveiled an unexplored role of fibroblasts as sentinel cells in the recruitment and activation of neutrophils to the initial site of the infection, an assumption that was confirmed by measurements of the levels of different cytokines and chemokines in the supernatant of *in vitro* infected fibroblasts, as well as in sera from patients with Chagas disease.

## Results and discussion

### Global transcriptome profiling revealed a differential host cell response at late time points of the infection with CL Brener and CL-14

A comparative transcriptome analysis of the *in vitro* infection of HFF cells with two cloned *T*. *cruzi* strains, CL Brener and CL-14, identified differences in the expression of parasite genes involved with the differentiation from intracellular amastigotes to extracellular trypomastigotes [11]. We proposed that these differences, which may impact parasite survival and dissemination in the mammalian host, constitute the main factors responsible for the contrasting virulence phenotypes observed between the two parasite strains. Although similar infection rates occurred when HFF cells were exposed to CL Brener and CL-14 trypomastigotes, the numbers of trypomastigotes released in the culture supernatant are significant lower in CL-14 infected cells compared to the CL Brener infection [11]. Altogether, our previous studies indicated a deficiency in the CL-14 genetic program related to amastigote/trypomastigote differentiation [11]. Using the same RNA-Seq dataset generated from HFF cells infected in biological triplicates with both parasite strains as well as from uninfected cells described by Belew et al. [11], we analyzed the host gene transcriptome during the two time points of the infection compared to the transcriptome of uninfected cells. S1 Table shows the total number of reads generated from each library, with an average of 102 million reads for each cDNA library that passed quality filtering, as well as the percentages of reads mapped to the human reference genome. It is noteworthy that, while at 60 hpi, the proportion of reads mapping to the human reference genome was similar for the infection with both strains (88.3% and 85.1% for CL Brener and CL-14 infection, respectively), at 96 hpi, this proportion was smaller for cells infected with CL Brener (62.2%) than for cells infected with CL-14 (86.8%). As discussed previously, this difference may reflect the difference in the number of intracellular amastigotes, which is higher at 96 hpi with CL Brener than with CL-14 [11]. Reads from uninfected cells showed a percentage of sequences mapping to the human reference genome greater than 99% (99.3% and 99.5% respectively for cells cultured for 48 and 60 hours).

**Fig 1:**
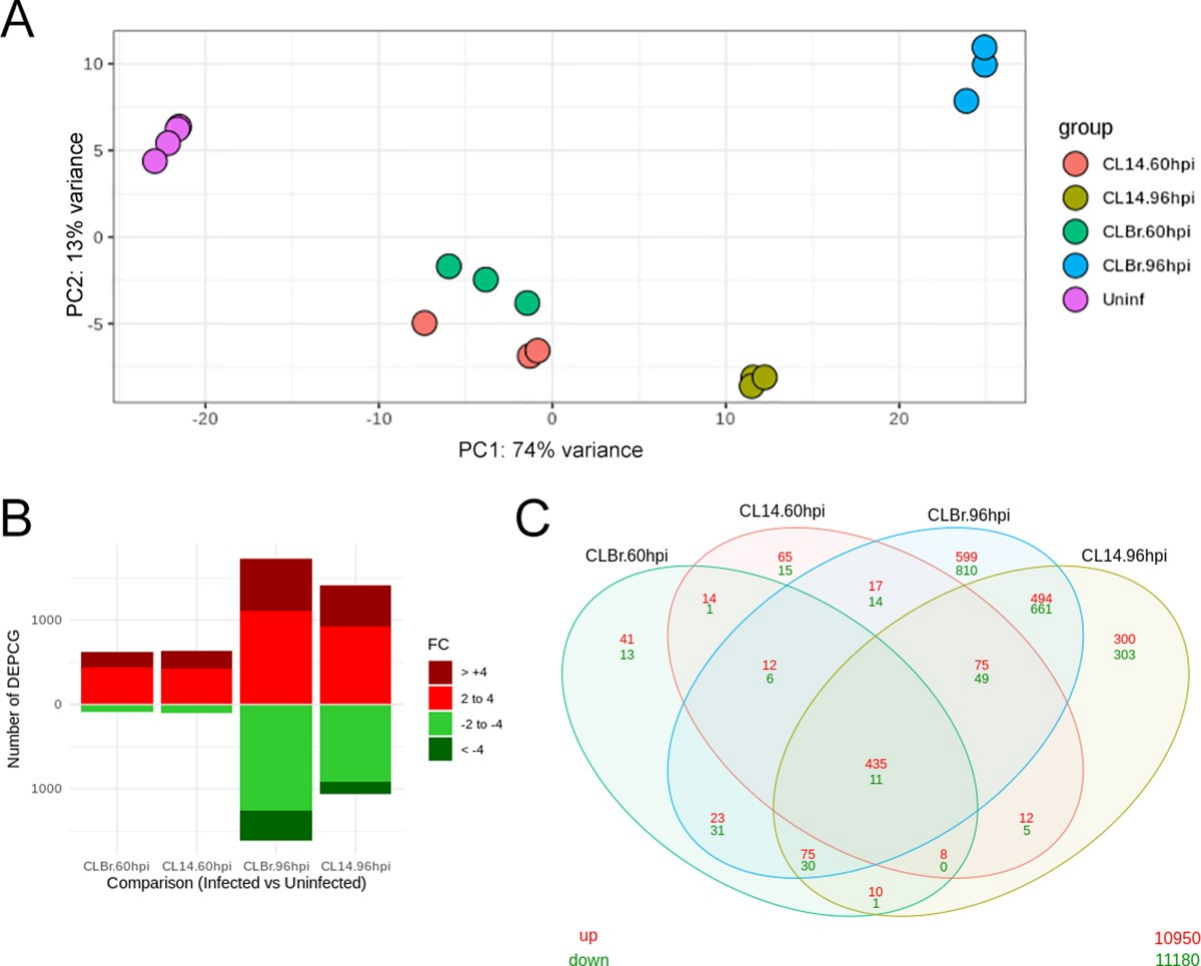
RNA-Seq data analysis and differential gene expression across human cells infected with T. cruzi CL Brener and CL-14. (A) Principal component analysis (PCA) plot from RNA-Seq data generated from HFF cells mapped to human genome. Each group was represented in different colors. (B) Bar plots of the numbers of protein-coding genes considered significantly differently expressed (adjusted p-value < .05) comparing infected cells vs uninfected samples. The numbers of genes in each category were defined as: fold changes 2–4 (light red for positive, light green for negative) and fold change >4 (dark red for positive and dark green for negative). (C) A Venn diagram analysis displays overlaps of upregulated (red) and downregulated (green) protein-coding genes among the different comparisons performed against infected and uninfected samples.

In order to inspect the relationships between samples, to assess reproducibility and to identify potential outliers, reads derived from all sixteen libraries that were mapped to the human genome and submitted to counts normalization (S2 Table) were analyzed using principal component analysis (PCA) and hierarchical clustering. As shown in Fig 1A, a high degree of similarity between the biological replicates can be inferred from clustering of replicate libraries observed in the PCA plot. Reads derived from uninfected cells cultured for 48 and 60 hours clustered together on the left side, with 74% of the variance explained by PC1 (Fig 1A), indicating that these cells display small changes during both time points in culture. Similarly, samples derived from cells infected for 60 hours with CL Brener and CL-14 also clustered together, indicating that similar changes in the global transcriptome profiles occurred during the initial phase of infection with either strain. In contrast, cells infected for 96 hours with CL-14 have a transcriptome profile that falls between the transcriptome of cells infected for 60 hours and CL Brener infected cells at 96 hpi. At the right end of the PCA plot, clustering of the three samples derived from cells infected for 96 hours with CL Brener indicated the existence of major differences between the host cell response to CL Brener and CL-14 at later time points of infection. Similar results are observed in the heatmap of hierarchical clustering, which showed a pattern that not only indicates the clustering between all biological triplicates but also a more pronounced difference between the host cell response to infection with the two strains at 96 hpi compared to 60 hpi (S1 Fig).

To obtain a detailed picture of the host cell response to the infection by the two parasite strains, we analyzed all differentially expressed protein coding genes (DEPCGs) as well as differentially expressed long noncoding RNAs (DElncRNAs). S3 Table shows tall DEPCGs and DElncRNAs that were identified in pairwise comparisons between infected and uninfected samples (with adjusted P value less than 0.05 and a fold change ≥ 2). In contrast to the small differences in the host cell responses observed at 60 hpi with both strains compared to uninfected cells (739 DEPCGs in CL Brener infection and 711 DEPCGs in CL-14 infection), at 96 hpi, the numbers of protein coding genes presenting a fold change greater than 2 are significantly higher in CL Brener infection (3342 DEPCGs, 1730 upregulated and 1612 downregulated) compared to CL-14 infected cells (2469 DEPCGs, 1409 upregulated and 1060 downregulated) (Fig 1B and Table 1). The Venn diagram in Fig 1C also shows that, while at 60 hpi, 54 genes were differentially expressed exclusively in the CL Brener infection and 80 DEPCGs were identified only in the CL-14 infection, these numbers increased up to 1409 genes at 96 hpi with CL Brener, i.e., changes in the expression of 1409 genes occur exclusively in CL Brener infected cells at late time points while only 603 host genes were identified as exclusively DEPCGs at 96 hpi with CL-14. Thus, these results indicated that, similarly to the changes observed in the transcriptome of parasite genes [11], at later time points, the host cell responds more drastically to the infection with a virulent strain compared to an avirulent one (Fig 1C). It can be also inferred that the differences observed in the transcriptome profiling of host protein coding genes is not related to differences in the capacity of each strain to establish an intracellular infection, or in the capacity of the host cell to respond to the initial phase of infection. In contrast, because CL-14 has a defective capacity to complete the final steps of the intracellular infection cycle, changes in the host cell response became less prominent during later time points of infection with CL-14 compared to CL Brener. Pairwise comparisons of DEPCGs between CL Brener and CL-14 infected cells again confirmed that at 96 hpi more human genes have differential expression in response to the infection with CL Brener compared to the CL-14 infection (S3 Table).

**Table 1:** Differently expressed protein coding genes between infected and uninfected samples.

	CL Brener 60 hpi	CL Brener 96 hpi	CL-14 60 hpi	CL-14 96 hpi
DEPCGs	711	3342	739	2469
Up	618	1730	638	1409
Down	93	1612	101	1060

### Genes related to immune response are among those displaying increased expression in response to infection with different *T*. *cruzi* strains

Previous RNA-Seq studies on the infection of human fibroblasts with the *T*. *cruzi* Y strain also showed very few changes in gene expression at earlier time points of the infection but increased numbers of differentially expressed host genes at 72 hpi [27]. During this late time point, a total of 1176 genes presented a fold change ≥ 2, whereas 450 and 1459 genes were identified as differentially expressed at 4 hpi and 24 hpi, respectively [27]. Also, the lack of major transcriptome differences at the host cell level observed in the *in vitro* model of infection with CL Brener and Y strain indicated that a common set of host genes are programmed to respond to the infection by virulent *T*. *cruzi* strains. Similar to the results observed during the comparison between CL Brener and CL-14 strains (Table 1; Fig 1C), the vast majority of DEPCGs are up-regulated in response to infection with the Y strain [27]. Also consistent with previous RNA-Seq studies with other parasite strains [27,28], most genes that were highly up-regulated in HFF cells upon infection with CL Brener and CL-14 strains are associated with the immune response, particularly, type I IFN response. At 60 and 96 hpi, among the highly upregulated genes in cells infected with CL Brener and CL-14 strains are genes encoding the chemokines CCL5, CXCL10 and CXCL11, the interleukins IL6 and IL8, interferon 1 beta (IFNB1), tumor necrosis factor (TNFSF10/TRAIL), the colony stimulating factor (CSF3) as well as others innate immune response related genes as RSAD2, OAS1, OAS2, OAS3 and OASL (S2 Fig). Li *et al*. [27] and Houston-Ludlam *et al*. [28] found similar up-regulation of the expression of immune related genes CXCL10, CXCL11, TNFSF10/TRAIL, RSAD2, OAS1, OAS2, OAS3, OASL at 72 hpi of HFF with *T*. *cruzi* Y or Sylvio strains compared with uninfected cells.

**Fig 2:**
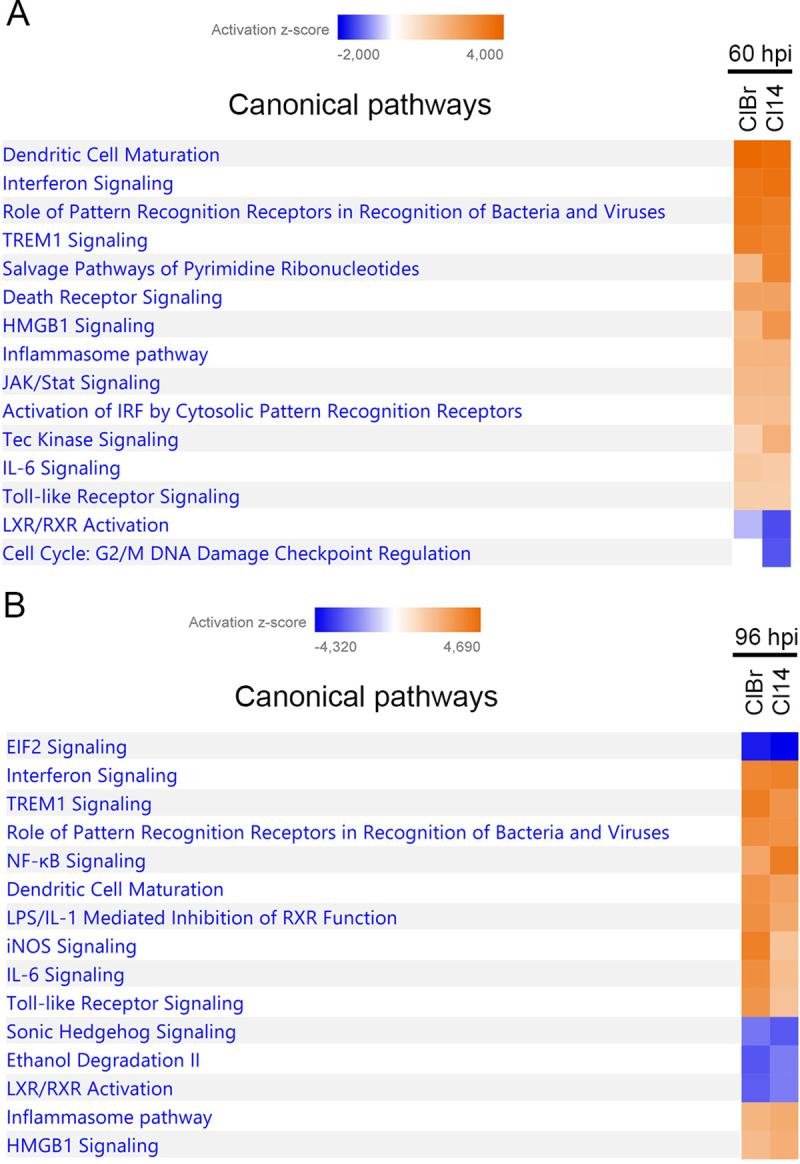
Enriched canonical pathways during *T*. *cruzi* infection at 60 and 96 hpi. The comparison of top 15 enriched canonical pathways to protein-coding genes during the infection with CL Brener and CL-14 at 60 hpi (A) and at 96 hpi (B) are displayed by their z-score. The CL Brener infection was represented by squares on the left and CL-14 by squares on the right. Enriched upregulated pathways was colored in a grade scale of orange, while downregulated pathways are colored in a grade scale of blue.

Using the Ingenuity Pathway Analysis (IPA) software, we performed enriched canonical pathways analyses to identify the cellular pathways associated with differentially expressed genes in response to infection with CL Brener and CL-14 strains (S4 Table). Among the top 15 enriched pathways identified at 60 hpi with CL Brener or CL-14, the large majority is associated with immune responses (“Dendritic Cell Maturation”, “Interferon Signaling”, “Role of pattern recognition receptors in recognition of bacteria and virus”, “TREM-1 Signaling”, “Inflammasome pathway”, “IL-6 Signaling”) (Fig 2A). Upregulation of immune response related pathways such as “Interferon Signaling”, “Recognition of Bacterial and Virus Patterns” and “TREM1 Signaling” have been also described in previous studies of the host cell response during *in vitro* and *in vivo* infection with other *T*. *cruzi* strains including Dm28, G, Sylvio, Y and Brazil [15,16,19–21,23,27,28]. Fig 2 also showed that more pathways are downregulated at 96 hpi with both strains compared to 60 hpi and that more pronounced differences in the z-score were observed between the enriched pathways identified in cells infected with CL Brener and CL-14 infections at 96 hpi than in 60 hpi (Fig 2B). At 96 hpi, cells infected with both strains showed similar up-regulation of “Interferon Signaling”, “Role of pattern recognition receptors in recognition of bacteria and virus”, “Inflammasome pathway” and “HMGB1 Signaling” pathways. In contrast, “iNOS Signaling”, “IL6 Signaling” and “Toll-like Receptor Signaling” appeared to be more significantly up-regulated in CL Brener infected cells than in CL-14 infection, whereas “NF-kB Signaling’ appeared more significantly upregulated in CL-14 infected cells than in CL Brener infection.

### Chemokines/Cytokines genes are among differentially expressed host genes during infection with a virulent and avirulent *T*. *cruzi* strains

**Fig 3:**
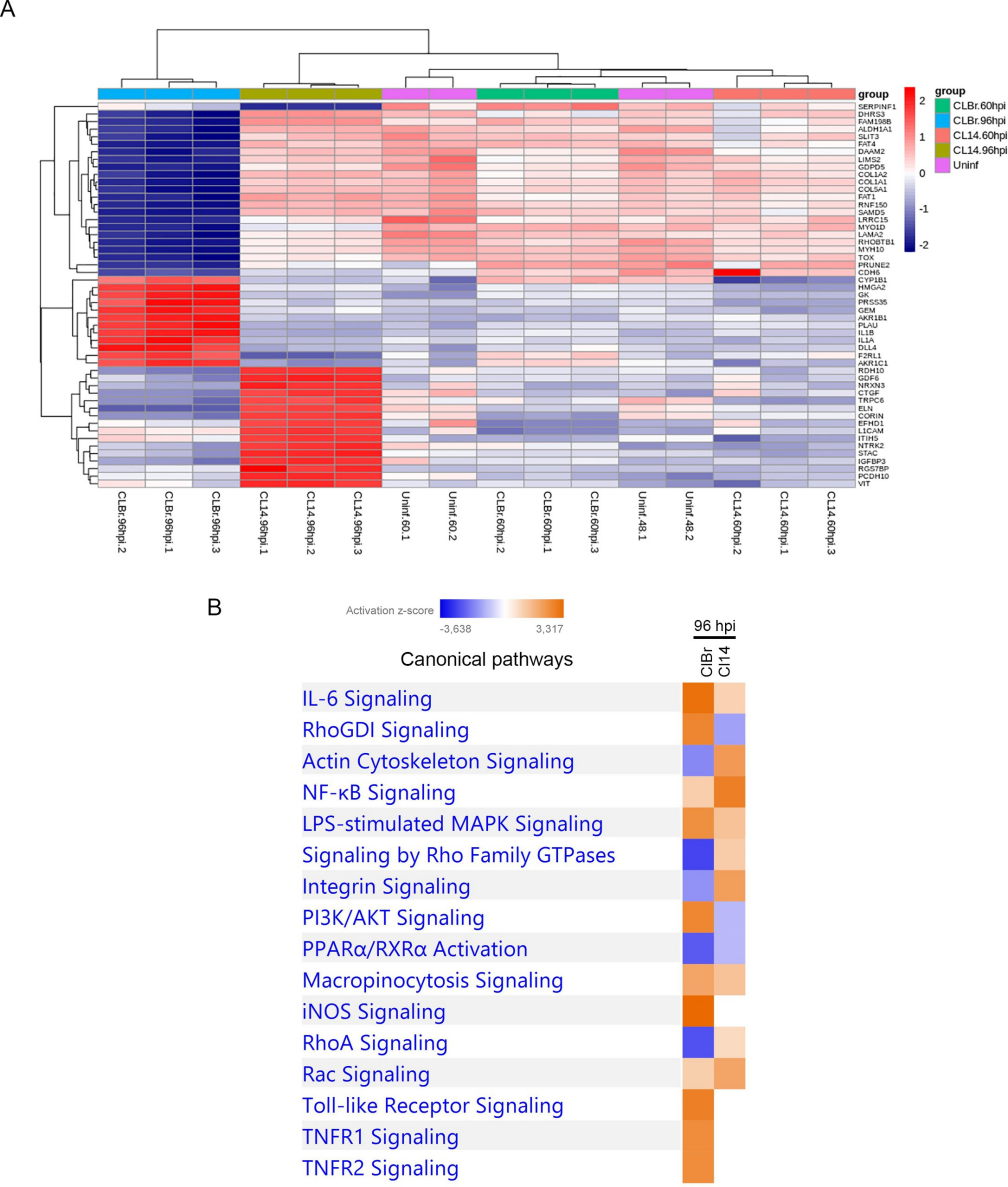
Enriched canonical pathways and heatmap from exclusive protein-coding genes during *T*. *cruzi* infection at 96 hpi. (A) An unsupervised clusterization analysis of top 51 exclusive protein-coding genes was performed to identify a gene cluster that represented the signature of human cell response during the infection with each *T*. *cruzi* strain. The most expressed genes were represented in a heatmap. (B) The comparison of top 16 enriched canonical pathways related to protein-coding genes during the infection with CL Brener and CL-14 at 96 hpi are displayed by their z-score. The CL Brener infection was represented at left and CL-14 at right. Enriched up-regulated pathways was colored in a grade scale of orange, while down-regulated pathways are colored in a grade scale of blue.

To further explore the differences in the human cell response against infection with a virulent and a non-virulent *T*. *cruzi* strain, we selected genes that were differentially expressed only during infection with CL Brener or CL-14 and performed clustering analyses of their normalized expression values (S3 Fig). As indicated above, at 96 hpi, 1409 DEPCGs were identified exclusively in cells infected with CL Brener compared to 603 genes that were differentially expressed exclusively in CL-14 infected cells (Fig 1C). The heatmap shown in Fig 3A revealed one cluster of genes whose expression is highly upregulated only in cells infected for 96 h with CL Brener and another cluster of genes significantly upregulated in cells infected with CL-14 at the same time point of infection. In addition, a group of genes are significantly downregulated only in cells infected with CL Brener. When these genes were analyzed with the IPA software, contrasting enrichment profiles during CL Brener and CL-14 infections were identified. As shown in Fig 3B, these analyses identified various pathways related to the immune response ("RhoGDI Signaling", “PI3k/AKT Signaling”, “TNFR1/2 Signaling”, “iNOS Signaling”, “IL6 Signaling” and “Toll-like Receptor Signaling”) as pathways that are highly upregulated, at 96 hpi, only in cells infected with CL Brener, while pathways related to cytoskeleton organization "Actin Cytoskeleton Signaling", "Integrin Signaling" and "RhoA Signaling" appeared as downregulated pathways only during CL Brener infection. The complete list of enriched pathways can be found in S4 Table. Previous studies on infection by different *T*. *cruzi* strains have also identified Toll-like Receptor Signaling and PI3k/AKT Signaling as upregulated pathways, as well as negative regulation of pathways related to cytoskeleton proteins [19–21,23,25,30,31]. It is noteworthy that upregulation of the PI3k/AKT Signaling, which has been identified in studies of RNAi knockdown as critical signaling pathway required for parasite replication, was observed only in cells infected with CL Brener [30]. On the other hand, a more pronounced upregulation of the NF-kB signaling pathway observed during the infection with CL-14 corroborates the results showing that host cell is more able to control the infection by the avirulent strain compared to the virulent one.

Notably, besides IL8 and IL-1β, we identified a group of chemokines such as CXCL5 and CXCL6, that were exclusively upregulated with a log2FC > 4 during CL Brener infection compared to uninfected controls (S2 Fig). The chemokines CXCL5 and CXCL6 have chemotactic and activating functions in neutrophils, especially during acute inflammatory responses [32]. In addition of having higher transcript levels of pro-inflammatory mediators such as CCL2, CSF3 and IL-6, genes encoding various inflammatory chemokines (CXCL1, CXCL2, CXCL3, CXCL5, CXCL6, CXCL8/IL8, CXCL10 and CXC11) have increased expression levels during the infection with CL Brener compared to the infection with CL-14. It is noteworthy that neutrophils are the major CXC chemokines responsive cell type, since they express CXCR1 and CXCR2 receptors, which are capable of binding to CXCL1, CXCL2, CXCL5, CXCL6, CXCL7 and CXCL8/IL8 [33].

### Co-expression networks built with coding and long non-coding host RNAs highlight differences in immune response signaling pathways during *T*. *cruzi* infection

To further characterize the pathways and gene networks that might be involved in the host cell response to *T*. *cruzi* infection, we selected from our RNA-Seq data all the differentially expressed polyadenylated lncRNAs (DElncRNAs) to build co-expression networks together with DEPCGs. About 82% of the identified DElncRNAs (see the complete list on S3 Table), were from two classes: large intergenic non-coding RNA (lincRNA) (~45%) and antisense lncRNAs (~37%) (S4 Fig). As shown in Table 2, the comparison between infected and non-infected HFFs at 60 and 96 hpi with both strains revealed similar numbers of DElncRNAs: 33 and 176, respectively, during the infection with CL Brener and 44 and 157 during the infection with CL-14. After analysing the transcriptome dataset generated by Li et al. [27], we identified a total of 26 and 73 DElncRNAs at 48 and 72 hpi, respectively (S5 Table). Thus, similar numbers of DElncRNAs were identified in the RNA-Seq data generated from the infection of the same human cell line with three different parasite strains, several of them being the same DElncRNAs.

**Table 2:** Differently expressed lncRNAs between infected and uninfected samples.

	CL Brener 60 hpi	CL Brener 96 hpi	CL-14 60 hpi	CL-14 96 hpi
DElncRNAs	33	176	44	157
Up	26	60	27	47
Down	7	116	17	110

**Fig 4:**
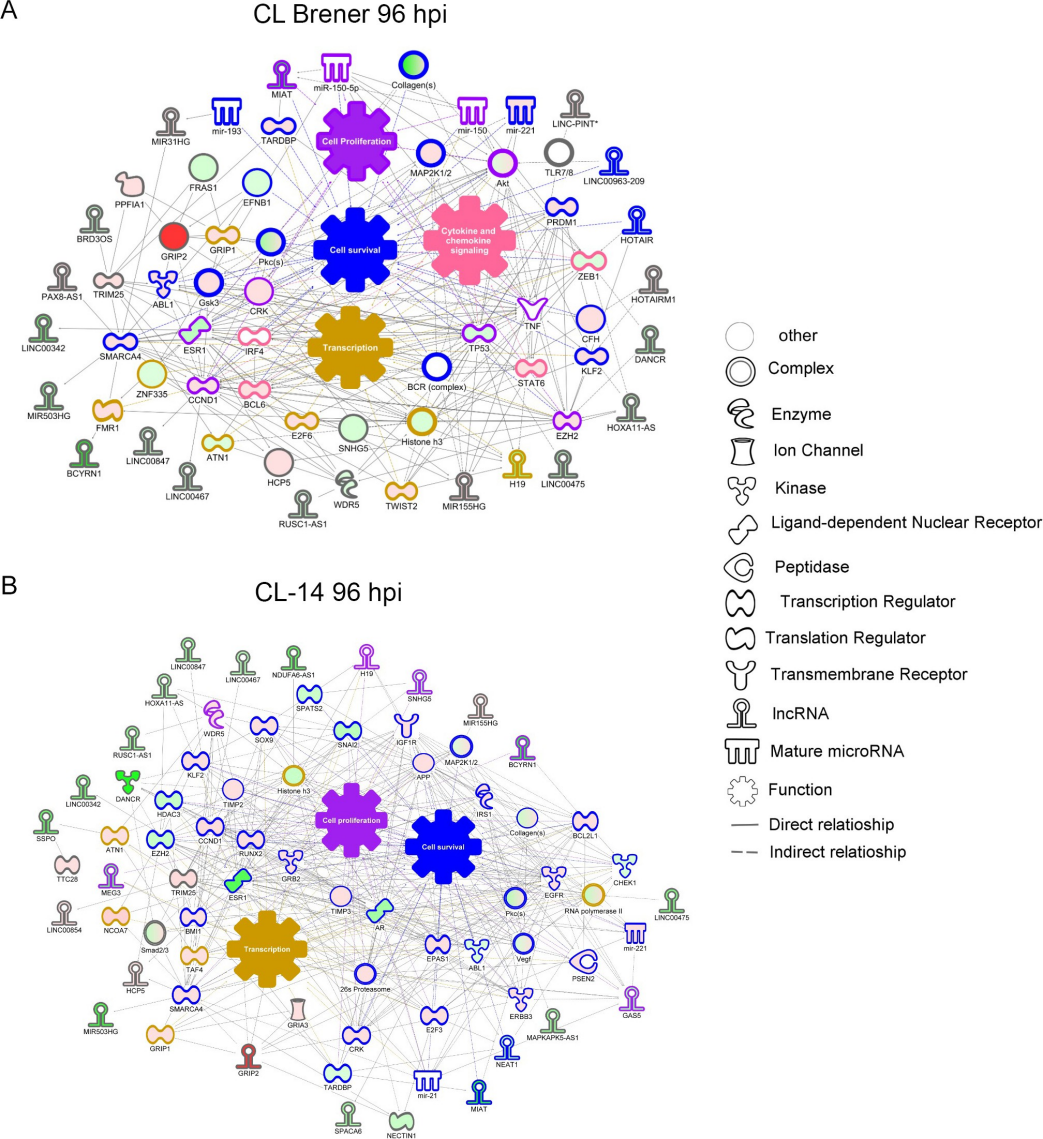
LncRNAs and protein-coding co-expression network interaction during *T*. *cruzi* CL Brener infection. A network of coding-lncRNAs during *T*. *cruzi* CL Brener (A) and CL-14 (B) infection showing their possible interaction. The nodes were represented in graduation of red (upregulated) and green (downregulated) based on their fold change in expression at 96 hpi. Each node shape represents one type of molecule as displayed in the legend.

To explore the role of these DElncRNAs as potential regulators of the host response to the infection by CL Brener and CL-14, we compared two co-expression networks that were built with DElncRNAs and DEPCGs derived from cells infected with the two strains. Both networks showed connections between genes related with cell survival, cell proliferation and transcription, but only for the infection with CL Brener we observed increased expression of genes related to cytokine/chemokine signaling (Fig 4). This pathway was connected to the upregulated lncRNA MIR155HG and the downregulated lncRNAs HOXA11-AS, H19, BCYRN1, DANCR, LINC00475, LIN00847, LINC00467, LINC00963-209. The lncRNAs DANCR, H19 and LINC00475 were also identified among the downregulated lncRNAs from the data generated by Li et al. [27]. The expression of some of these lncRNAs has been also investigated in other pathological conditions, such as the lncRNA LINC00475, whose expression is downregulated during dental pulp inflammation [34], as well as during infection of human primary pulmonary fibroblast (IMR-90) with adenovirus type 2 [35].

### Production of chemokines and cytokines by fibroblasts in response to infection by a virulent and avirulent *T*. *cruzi* strains

**Fig 5:**
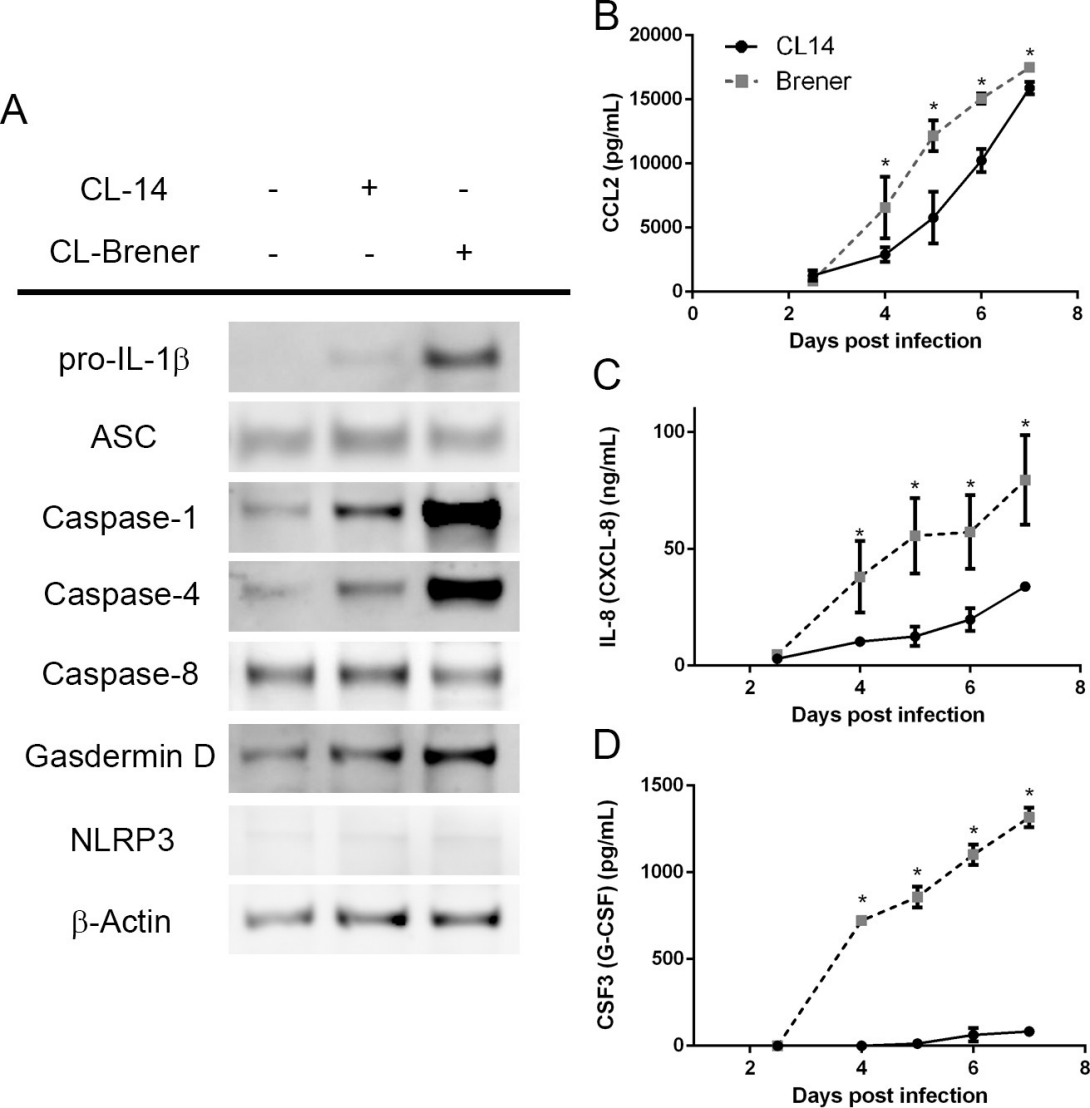
Differential cytokine and chemokine production by CL Brener and CL-14 infected fibroblasts. (A) Immunoblot of cell lysates from HFFs infected with *T*. *cruzi* CL Brener and CL-14 for 4 days. (B-D) Quantification of CCL2 (B), IL-8 (C), and CSF-3 (D) produced by HFFs in response to infection with *T*. *cruzi* CL Brener and CL-14 strains (10 parasites/cell, determined from 2.5 to 7 dpi). * p < 0.05 (unpaired t-test). (A) Image is representative of three independent experiments. (B-D) Data from three independent experiments (mean and s.e.m.).

As indicated in the previous sections, IL8, IL-1β and genes related to cytokine and chemokines signaling were among the major components of the host cell response that differentiates the infection by CL Brener and CL-14. To validate the results obtained from RNA-Seq analyses, we determine the levels of various chemokines and cytokines including CCL2, CSF3, IL8 and IL-1β in the supernatant HFF cells infected with CL Brener and CL-14 from 2.5 to 7 days post infection. We also measured the expression of the inflammasome components pro-caspase-1, caspase-4, and gasdermin D, all of which were found to be differentially upregulated accordingly to the RNA-Seq analysis. The inflammasome is a macromolecular complex assembled in response to a multitude of signals and culminates in the production of pro-inflammatory cytokines such as IL-1β via proteolysis of the precursor pro-IL-1β [36]. As shown in Fig 5A, immunoblot analyses of cell lysates at 4 days post-infection confirmed the enhanced expression of pro-IL-1β, with no evidence of the mature p10 IL-1β, which suggests that the inflammasome is not activated in this cell type at this time point. Accordingly, the caspases 1 and 4 were found to be highly upregulated in CL Brener-infected HFFs, whilst other inflammasome components such as caspase-8, ASC, and gasdermin D maintained comparable expression levels in uninfected and infected cells (Fig 5A), similar to a previous report studying murine myocardial tissue [37]. Interestingly, although increased levels of IL-1β mRNA were observed in CL-Brener infected cells, we were unable to detect this cytokine in culture supernatants from days 4 to 7 post-infection, indicating that *T*. *cruzi* does not activate the inflammasome in fibroblasts. According to our transcriptome analyses as well as protein data (Fig 5A), HFFs do not express NLRP3, the nod-like receptor responsible for inflammasome activation in the context of *T*. *cruzi* [37,38]. Thus, the absence of NLRP3 might explain the lack of mature IL-1β in the supernatants. In contrast, infection of THP-I macrophages with both *T*. *cruzi* strains resulted in high levels of IL-1β (S5 Fig), which is generated via NLRP3 [39].

Similar to IL-1β, although transcript levels of other cytokines and chemokines were upregulated in cells infected with both strains, we were not able to detect the expression of CCL8, CCL20, CXCL10 and IL33 in the supernatant of HFF cells infected with CL Brener or CL-14. In contrast, CCL2, IL-8 and CSF3 were detected in the supernatant of cells infected with both parasite strains (Fig 5B–5D). To better understand whether CL-14-infected fibroblasts have a delayed production of these cytokines, we investigated the presence of these molecules in the supernatants up to 7 dpi. Although the levels of CCL2 are significantly lower in the supernatant of HFF cells infected with CL-14 on days 4, 5 and 6 post-infection compared to CL Brener infected cells, at day 7, similar levels of this chemokine were detected in the supernatant of HFFs infected with both strains (Fig 5B). CCL2 is a chemokine produced by a diversity of cells in response of inflammation and act as a critical factor in the recruitment of immune cells such as neutrophils, lymphocytes, and monocytes [40–42]. Paiva et al. [43] have shown that CCL2 also contributes to reduce parasite growth by controlling the distribution, cellular composition and state of activation of inflammatory infiltrates during acute *T*. *cruzi* infection. Different from CCL2, significantly higher levels of IL-8 and CSF3 were detected in the supernatants of cells infected with CL Brener compared to CL-14 at all time points of the infection (Fig 5C and 5D). CSF3, also known as granulocyte colony-stimulating factor (G-CSF), is a major regulator of neutrophilic granulocytes [44]. It was suggested that this cytokine induces immunomodulation, recruitment of T regulatory cells, reduction of myocarditis and decrease of parasite load in a mouse model of chronic Chagas disease cardiomyopathy [45]. Both CL-14 and CL Brener infection stimulated HFF cells to produce IL8 in the nanomolar range, although its levels are significantly higher in CL Brener infected cells compared to Cl-14 infected cells throughout the course of infection (Fig 5C). As discussed before, differences in IL8 expression were also identified during infection of Sylvio and Y strains infected HFF cells [28]. IL8 acts both as a chemotactic factor and as stimulatory factor for neutrophils [46,47]. Importantly, the presence of these cytokines in the supernatant precedes parasite release, as previously shown [11] and cell death, as shown in S6 Fig, making it less likely that cellular damage in directly involved in the production of these cytokines up to 4 dpi.

Immunohistochemical analyses of cutaneous tissues from *T*. *cruzi* infected mice identified neutrophils at 2 hpi and confirmed the presence of macrophages and neutrophils at 24 hpi compared to uninfected mice [19]. Although the role of fibroblast in neutrophil recruitment during *T*. *cruzi* infection has not yet been investigated, several other studies showed that fibroblast may act in neutrophil recruitment and activation under inflammatory conditions [48]. Interestingly, IL8 and CSF3 act synergistically to promote neutrophil activation [47]. Together with the scarcity of studies addressing the role of fibroblasts in neutrophil recruitment during *T*. *cruzi* infection, our RNA-Seq data and ELISA results led us to investigate a potential role for human fibroblast on neutrophil activation.

### Neutrophils are recruited and activated by *T*. *cruzi*-infected HFF with both parasite strains

**Fig 6:**
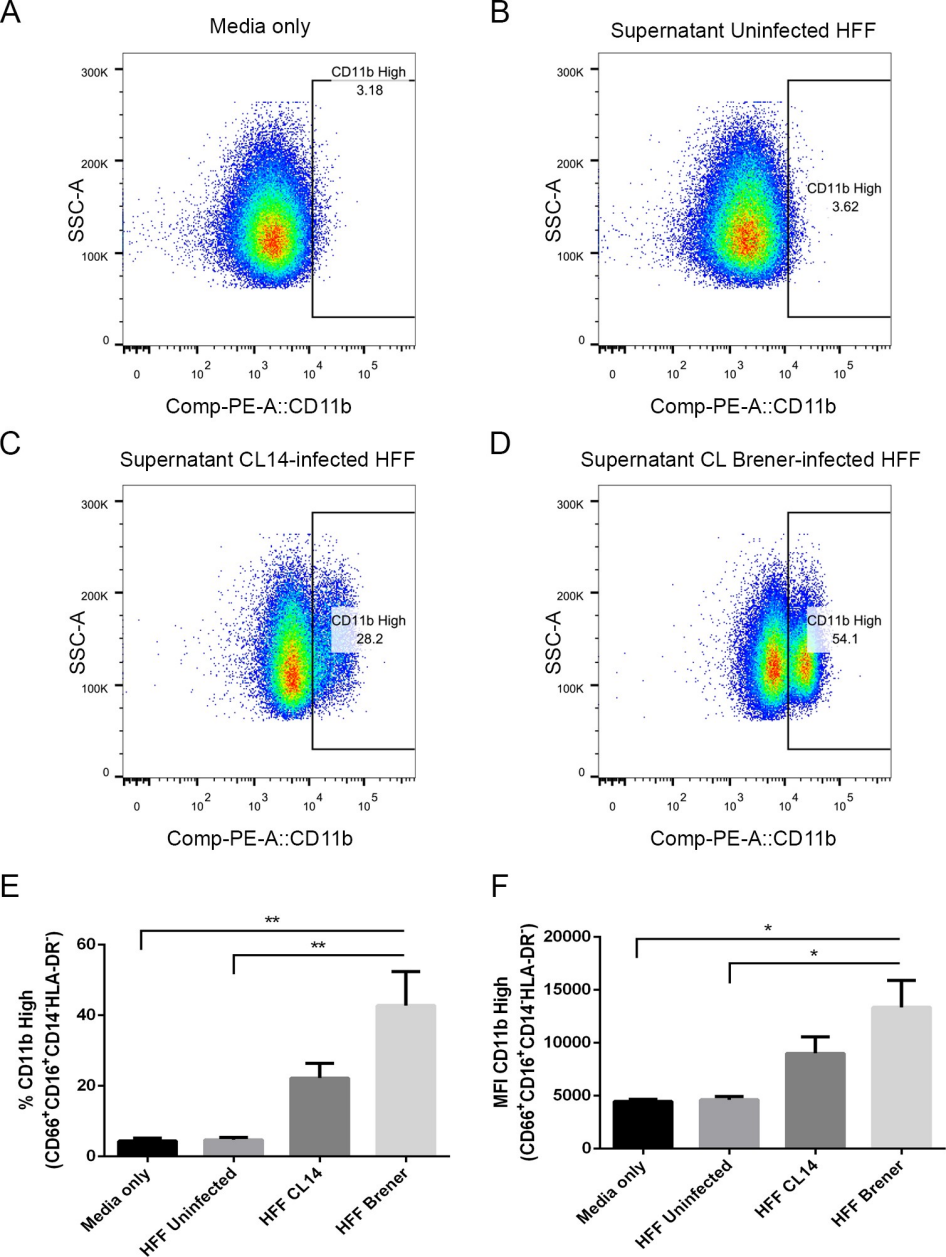
Human neutrophils incubated with supernatants from *T*. *cruzi*-infected HFF cells have enhanced expression of CD11b. Flow cytometry analysis of live neutrophils (CD16^+^CD66b^+^CD14^-^HLA-DR^-^) incubated for 16 hours with media only (A), supernatants from uninfected HFF cells (B), or supernatants of HFF cells infected for 4 days with *T*. *cruzi* CL-14 (C) or CL Brener (D). Percentage of cells expressing high levels of the activation marker CD11b (E) and mean fluorescent intensity (MFI) of CD11b (F). * p < 0.05, ** p < 0.01 (one-way ANOVA with Tukey’s post-test comparing the indicated treatments). (A-D) Images are representative of three independent experiments. (E-F) Data from three independent experiments (mean and s.e.m.).

The results presented above indicated that, upon infection with *T*. *cruzi*, human fibroblasts modulate the activity of immune cells such as neutrophils. To further investigate this activity, we collected human neutrophils from healthy donors and incubated them with supernatants from infected HFFs at 4 dpi. Twenty-four hours later, the expression levels of CD11b in the live CD16^+^CD66b^+^CD14^-^HLA-DR^-^ population were quantified by flow cytometry (gating strategy can be found in S7 Fig). CD11b is constitutively expressed in human neutrophils, and has its expression enhanced upon activation, allowing it to be used as an activation marker [49]. Incubation with supernatants from uninfected HFF cells did not cause an increase in CD11b expression in comparison to controls incubated with fresh media only (Fig 6A and 6B). CD11b expression is, however, enhanced upon incubation with supernatants from CL Brener-infected or CL-14-infected HFFs. Corroborating the results showing the production of different cytokine/chemokynes, supernatants from *T*. *cruzi*-infected fibroblasts resulted in increased CD11b expression and a subpopulation in which 29.9% or 48.7 of the cells displayed high expression of CD11b depending on the parasite strain (Fig 6A–6F). These data suggest that, as a result of the enhanced expression of cytokines and chemokines by fibroblasts upon infection with distinct *T*. *cruzi* strains, neutrophils were activated, as illustrated by the high levels of CD11b. The finding that both G-CSF and IL-8 induce enhanced CD11b expression is supportive of this hypothesis (S8 Fig). Our results also indicated that cells infected with the more virulent parasite strain, CL Brener, have increased capacity to secrete cytokine/chemokynes that activate neutrophils in response to infection.

**Fig 7:**
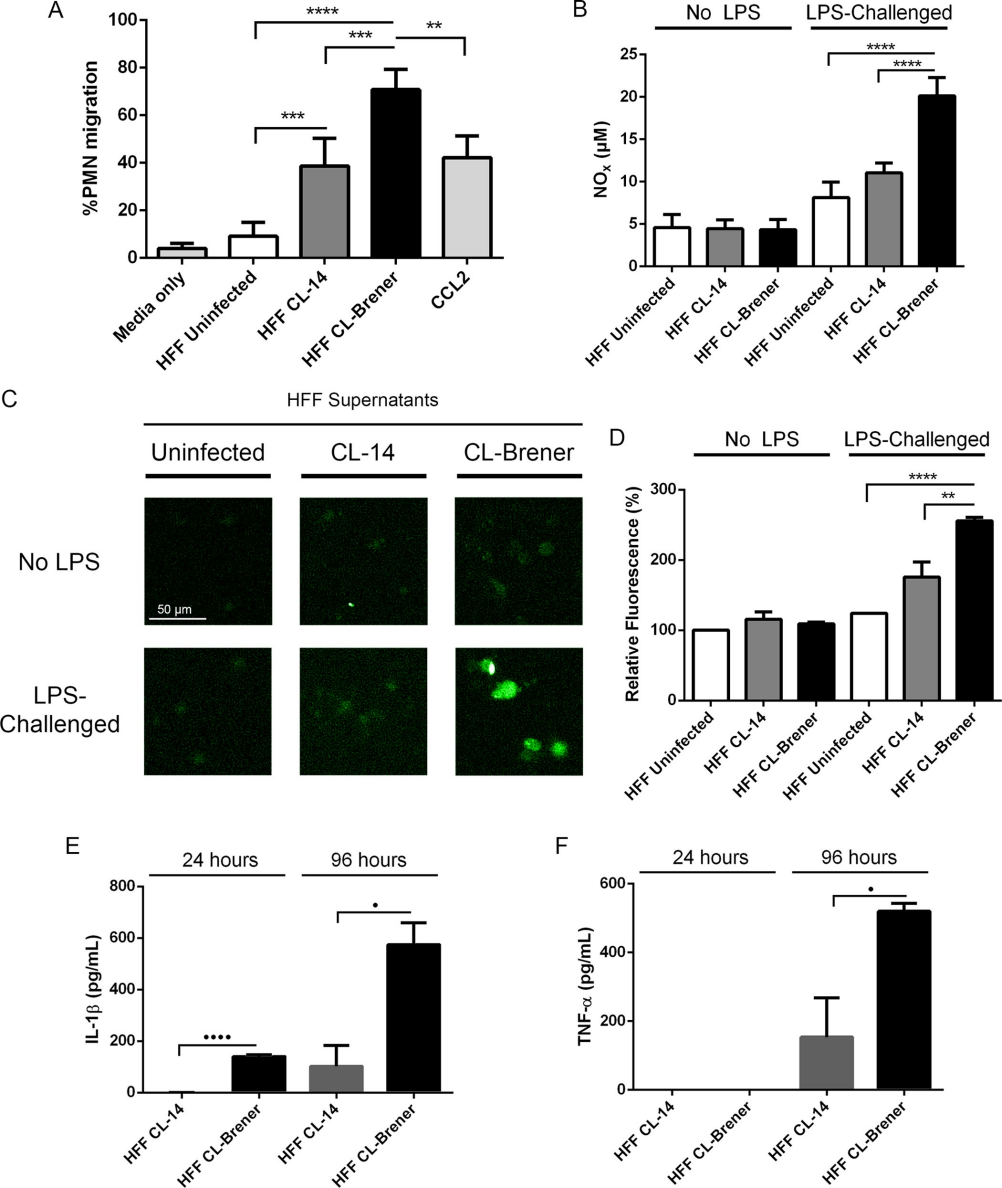
Human neutrophils are activated by supernatants from *T*. *cruzi*-infected HFF cells. (A) Migration assays of neutrophils incubated for 16 hours with media only, supernatants from uninfected HFF cells, supernatants of HFF cells infected for 4 days with *T*. *cruzi* CL-14 or CL Brener, and with purified CCL2 (500 ng/mL). (B-D) NO (B) and ROS assays (C-D) of neutrophils incubated for 16 hours with media only, supernatants from uninfected HFF cells, supernatants of HFF cells infected for 4 days with *T*. *cruzi* CL-14 or CL Brener and then challenged with 200 ng/mL LPS for one hour. IL-1β (E) and TNF-α (F) production in PMNs incubated for 24 and 96 hours with supernatants of HFF cells infected for 4 days with *T*. *cruzi* CL-14 or CL Brener. No TNF-α nor IL-1β detected in PMNs treated with media only or supernatants from uninfected HFF. * p < 0.05, ** p < 0.01, *** p < 0.001, and **** p < 0.0001 (one-way ANOVA with Tukey’s post-test comparing the indicated treatments). • p < 0.05 and •••• p < 0.0001 (unpaired t-test comparing the indicated treatments). (C) Image is representative of three independent experiments. (A) Data from four independent experiments (mean and s.e.m.). (B, D-F) Data from three independent experiments (mean and s.e.m).

Next, we focused on the characterization of several neutrophil biological activities that might be influenced by the *T*. *cruzi*-infected fibroblasts. To correlate the production of chemokynes with the recruitment of immune cells, cell migration assays were performed using human neutrophils. In these assays, a lower chamber was loaded with supernatants from HFF-infected (4 dpi) or uninfected cells, as well as fresh media (negative control) or CCL2 (positive control). Neutrophils were then added to an upper chamber, separated from the lower chamber by a 5 μm porous membrane and, after 1.5 h incubation, the number of neutrophils in the lower chamber was quantified. While fresh media or supernatants from uninfected HFF induced very little migration (lower than 10%), supernatants from HFF-infected cells induced a much higher neutrophil migration, and once again, this response was more pronounced in neutrophils incubated with supernatants from CL Brener-infected HFFs (Fig 7A). Neutrophils primed with supernatants from CL Brener-infected cells also showed enhanced production of NO and ROS (Fig 7B–7D), suggestive of enhanced neutrophil activation. We also analyzed the production of IL-1β and TNF-α, both potent pro-inflammatory cytokines relevant in *T*. *cruzi* infection [37,50,51]. Incubation of human neutrophils with media only or with uninfected HFF supernatants does not resulted in production of IL-1β and TNF-α. In contrast, supernatants of HFFs infected with both parasite strains stimulated IL-1β and TNF-α production by human neutrophils. As shown before, production of both cytokines occurs more efficiently when cells were stimulated with supernatants from CL Brener infected cells than with CL-14 infected fibroblasts (Fig 7E and 7F).

### CCL2, CSF3, IL-8 and IL-1β were increased in the sera of Chagas disease patients

**Fig 8:**
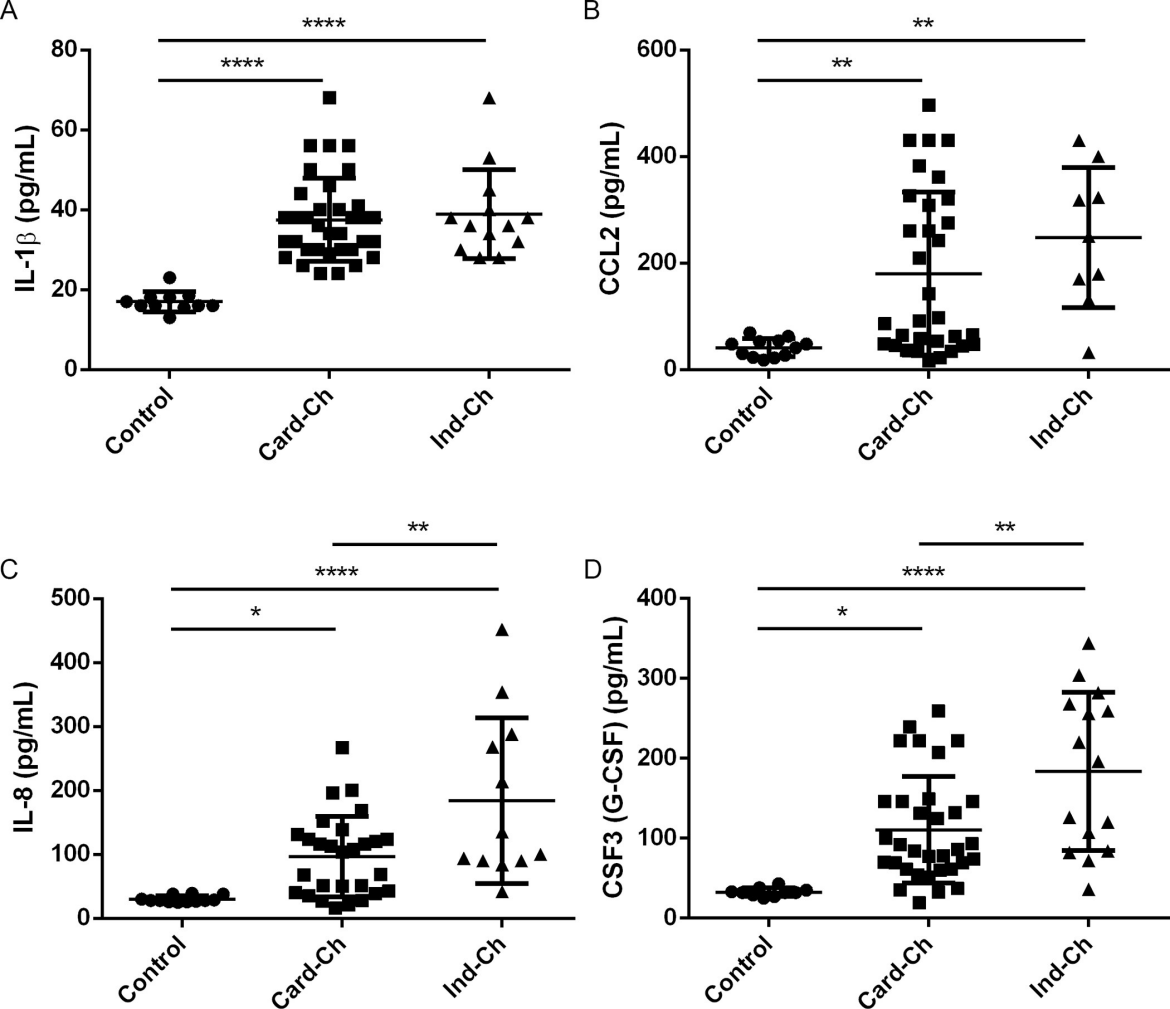
Cytokines and chemokines are enhanced in the serum of Chagas patients. Serum levels of IL-1β (A), CCL2 (B), IL-8 (C), and CSF3 (D) from cardiac (Card-Ch) and indeterminate (Ind-Ch) chagasic patients and healthy individuals (Control), as quantified by Bio-Plex assay. Number of individuals as follows: IL-1β: 11 control, 35 Card-Ch, 13 Ind-Ch. CCL2: 12 control, 28 Card-Ch, 12 Ind-Ch. IL-8: 10 control, 33 Card-Ch, 15 Ind-Ch. CSF3: 12 control, 32 Card-Ch, 9 Ind-Ch. ** p < 0.01. **** p < 0.0001. One-Way ANOVA with Tukey’s post-test. Bars represents standard deviation.

To investigate whether IL-1β, IL-8, CSF3, and CCL2 played a role in Chagas disease, we analyzed the levels of these cytokines and chemokines in plasma from patients diagnosed with the cardiac (Card-Ch) and indeterminate (Ind-Ch) forms of the disease as well as from health individuals. As shown in Fig 8, sera from both Card-Ch and Ind-Ch patients have increased levels of these molecules compared to control sera. Interestingly, whilst no difference between Card-Ch and Ind-Ch was observed regarding IL-1β and CCL2, the levels of both IL-8 and CSF3 were elevated in Ind-Ch when compared to Card-Ch (Fig 8). The source of these molecules *in vivo* is still unknow.

The chemokine CCL2 was previously linked to parasite burden and cell infiltration in murine models [43], and in accordance to our findings, sera of patients diagnosed with chronic cardiomyopathy also presented increased CCL2 levels in relation to health controls [52]. Additionally, that study also reported enhanced TNF-α levels in said patients. TNF-α is a potent pro-inflammatory cytokine, and its production might be linked to stimulated neutrophils as reported above (Fig 7F). IL-1β was linked to cardiomyocyte hypertrophy *in vitro* [51], and IL-1β gene polymorphism were correlated with susceptibility to Chagas cardiomyopathy in humans [53]. In our study, the virulent CL Brener strain induced enhanced pro-IL-1β expression in fibroblasts. Interestingly, we found no evidence of pro-IL-1β processing to its mature form IL-1β in this cell type. We did, however, found that supernatants from CL Brener-infected fibroblasts were able to stimulate neutrophils to produce IL-1β (Fig 7E). At this moment it is unclear whether fibroblasts can be a source of mature IL-1β *in vivo* in *T*. *cruzi*-infected individuals and if pro-IL-1β from fibroblasts has a biologically relevant role in this infection. This is, to our knowledge, the first study to report serum levels of IL-8 and CSF3 in Chagas disease patients. Our findings suggest that the increased levels of CCL2, IL-8, IL-1β, and CSF3 in humans infected with *T*. *cruzi* (Fig 8) might be directly and/or indirectly related to a previously overlooked role of fibroblasts as sentinel cells in the context of *T*. *cruzi* infection. Our study also suggests that the levels of these cytokines and chemokines in the plasma of Chagas disease patients may be dependent on the parasite strain.

Altogether, our data showed that human fibroblasts build a strong immune response upon infection by *T*. *cruzi* and that this response is differentially built depending on the parasite strain: infection with the virulent CL Brener strain induces a more robust inflammatory response compared with the CL-14 strain, which has an avirulent phenotype in animal models of infection. Our data also indicate that the mechanisms behind this genetic reprogramming may involve regulatory noncoding RNAs such as lncRNAs, known to modulate gene expression at the epigenetic, transcriptional, and post-transcriptional levels. As shown by our previous study based on the parasite transcriptome analysis of the same human cell line, the differences in the host response observed with the two parasite strains might be related to various parasite molecules that are also differentially expressed throughout the infection. More specifically, the distinct patterns observed regarding the expression of genes encoding parasite surface proteins that are associated with the differentiation from replicative amastigotes to trypomastigotes, which occurs during latter time points of the infection, may be one of the factors responsible for the differences in host cell response described here. It is also possible that differences observed in gene expression simply reflect the delayed development of trypomastigotes and parasite egress that it is observed with the CL-14 strain compared to CL Brener [11]. Irrespective of these differences, one of the consequences of the genetic reprogramming of fibroblast upon infection by *T*. *cruzi* is the recruitment and activation of immune cells such as neutrophils. Again, this recruitment may occur differently depending on the virulence phenotype of the parasite strain. Human dermal fibroblasts are one of the first cells to be infected by the trypomastigotes released on the skin by an infected triatomine vector. We hypothesize that fibroblasts initiate and modulate a potent innate immune response, resulting in enhanced neutrophil activity at the initial site of the infection. Together with differences in the host genetic background, such early host immune response, which varies depending on the parasite strain, may play a pivotal role in determining the outcome of Chagas disease.

## Material and methods

### Parasite cultures, cell infection, RNA extraction and cDNA sequencing

Parasite cultures, cell infection, RNA extraction and cDNA sequencing were performed as previously described [11]. Briefly, tissue culture derived trypomastigotes from the two cloned *T*. *cruzi* strains, CL Brener and CL-14, were used to infect sub-confluent monolayers of human foreskin fibroblasts (HFF; ATCC CRL-2522) at a ratio of 80 parasites: host cell. Total RNA was extracted from infected HFF at 60 and 96 hours post-infection using the Trizol reagent (Invitrogen, CA, USA). After DNAse treatment, the RNA was further purified using the Qiagen RNeasy mini kit, poly(A)+ RNA was selected using oligo-dT following Illumina TruSeqv2 instructions and its integrity was assessed using an Agilent 2100 bioanalyzer. cDNA libraries were constructed using a TruSeq RNA Sample Prep Kit Version II (Illumina) with an average insert size of ~300 nt. Libraries were sequenced on an Illumina HiSeq 1500. For uninfected samples, total RNA was extracted from HFF cells cultured for 48 and 60 hours and RNA extraction and cDNA sequencing were performed using the same protocols.

### Data quality assessment and visualization

Quality assessment of 100 nucleotide paired-end reads was performed via an initial evaluation with FastQC (http://www.bioinformatics.babraham.ac.uk/projects/fastqc/) and biopieces (http://biopieces.org). Illumina sequencing adapters and low quality sequences were removed with Trimmomatic [54]. The remaining sequences from infected and uninfected samples were mapped using TopHat2 [55] version 2.0.14 in the human genome ensembl GRCh37.62.v3 release and options allowing a single randomly placed mapping per read (-g 1) and without a novel splice junction search (-G gff_file). Alignments were compressed, sorted, and indexed using samtools [56] in order to include only proper pairs and counted against the set of annotated transcripts using HTSeq [57]. The resulting count tables were passed to R script for outlier testing, sample clustering, visualization, and differential expression analyses. A table with raw counts for all samples and conditions is available as supplementary data (S2 Table). RNA-seq data were deposited at the National Center for Biotechnology (NCBI) Sequence Read Arquive (SRA) under bioproject PRJNA389926.

### Differential expression, canonical pathways and network-interaction

Samples were tested for significant outliers using a mix of hierarchical clustering and principal component analysis. Non-expressed and weakly expressed genes, defined as having <2 counts per million in n of the samples, where n is the size of the smallest group of replicates (here n = 2), were removed prior to differential expression (DE) analysis. Of the 51,041 genes analyzed, 13,130 remained after applying the low-count filter. Data normalization and the differential expression analyses were performed using DESeq2 [58]. Genes were considered ‘significant’ when the |log2 fold-change| was greater than 1.0 (logFC>1) and the adjusted P value was less than 0.05. The annotation for each gene was obtained by biomart [59] accessing the information about the human genome version hg19/GRCh37. The differentially expressed genes were separated in protein-coding and non-coding genes by the biotype annotation. Genes without a biotype identification were removed from further analyses. The set of significant protein coding genes for each contrast of interest was analyzed in Ingenuity Pathway Analysis (IPA) software to identify the enriched canonical pathways, as well the network-interaction between lncRNAs and protein-coding genes.

### Chemokines and Cytokines quantification and LDH activity

The concentration of cytokines and chemokines in the culture supernatants from HFF cells cultivated *in vitro* and infected by CL Brener and CL-14 at 4 dpi (10 parasites/cell) were assessed by ELISA following the manufacturer’s instruction. Briefly, 10^5^ HFF cells suspended in DMEM supplemented with 10% fetal bovine serum were added to a well (in 24-wells plate), and maintained overnight at 37 ^o^C and 5% CO_2_. After infection with CL-14 and CL Brener for 4 hours, the cells were washed three times in fresh media to remove non-adherent parasites. After the indicated time points, the supernatants were collected, centrifuged (12,000 x G for 10 minutes, at 4 ^o^C) to remove cellular debris and parasites, and the clean supernatant was used to quantify LDH release and cytokine production. The production of TNF-α and IL-1β by neutrophils was assayed by ELISA after 24 and 96 hours stimulation with media from HFF-infected cells. All kits were manufactured by R&D Systems, and their catalogue numbers are as follows: CCL2 (DY279), CCL8 (DY281), CCL20 (DY360), CXCL10 (DY266), CSF3 (DY214), IL1β (DY201), IL8 (DY208), IL33 (DY3625B) and TNF-α (DY210). For CCL2, IL1β, IL8 and CSF3 the supernatants were collected until 7 dpi. LDH activity was assayed using CytoTox 96 Non-Radioactive Cytotoxicity Assay (Promega), following the manufacturer’s instructions, and using non-infected HFFs maintained in the same conditions, lysed in 0.8% Triton X-100, as 100% LDH activity.

### Immunoblot assays

After infection for 4 days as described above, HFFs were washed 3 times with 4 ^o^C PBS, and harvested using 0.25% trypsin (GIBCO). Following 3 more washes with PBS, the cells were lysed for 10 minutes at 4 ^o^C using RIPA Buffer (Sigma) supplemented with Roche cOmplete protease inhibitor cocktail (Merck), and the protein fraction was collected from the supernatants after centrifugation (13,000*g*, 4 ^o^C, 10 minutes). The proteins were then subjected to immunoblotting and probed with the following antibodies: IL-1β (R&D Systems, MAB601, 1:1000 dilution), ASC (Santa Cruz, sc-271054, 1:100 dilution), caspase-1 (Adipogen, AG-20B, 1:500), caspase-4 (Cell Signaling, 44505, 1:1000), caspase-8 (Cell Signaling, 94965, 1:1000), gasdermin D (Cell Signaling, 964585, 1:1000), NLRP3 (R&D Systems, MAB7578, 1:100), and β-actin (Sigma, A2066, 1:2000). Uncropped images of immunoblots are provided as S9 Fig.

### Neutrophil purification

PMN cells were isolated from peripheral blood from healthy adults following protocols approved by the Ethical Committee from the Centro de Pesquisa Rene Rachou (COEP-CPqRR 665281). All adult subjects signed a written informed consent. Pregnant women and children were not enrolled in the study. Briefly, blood was gently added to a 50 mL conical tube containing 15 mL of Ficoll (Sigma-Aldrich) and then centrifuged at 400 x G (with slow acceleration and deacceleration) for 40 min. at room temperature. Next, the interphase containing PMN was collected, transferred to a new tube, and contaminating red blood cells were lysed using ACK buffer (Ammonium-Clhoride-Potassium Buffer, Thermo Fisher) for 10 minutes at 4 ^o^C, followed by centrifugation at 400 x G for 8 min. at 4 ^o^C. The supernatant was then discarded, and the pellet subjected to a second round of ACK lysis and centrifugation. After successive washes with PBS, the PMN pellet was resuspended in RPMI 1640 supplemented with 10% FBS, and the cell density quantified using a hemocytometer. To confirm the quality of the preparation, an aliquot of the purified PMN was fixed and stained according to the manufacturer’s instructions (Shandon Kwik-Diff Stains, Thermo Fisher) (S10 Fig).

### Flow cytometry

2x10^6^ PMNs purified as described above were incubated for 16 hours with supernatants from HFF cells (uninfected or CL-14 or CL Brener-infected, collected at 4 dpi). After incubation, cells were washed with PBS, and dead cells were stained using a AmCyan Live/Dead kit as described by the manufacturer (Thermo Fisher, L34957). Next, Fc receptors were blocked by incubating the cells with Human FcR blocking reagent (Miltenyi Biotec, cat 120-00-442) for 20 minutes at 4 ^o^C. After wash with FACS buffer (PBS containing 10% FBS), the following receptors were stained by incubating then for 30 min. at 4 ^o^C: CD14 APC-eFluor 450 (1:100, eBioscience, cat 48-0149-42), CD16 PeCy7 (1:100, BD, cat 557744), HLA-DR eFluor 780 (1:100, eBioscience, 47-9956-42), CD66b-FITC (1:100, BD, cat 555724), and CD11b FITC (1:100, BD, cat 555388). After one more wash, cells were resuspended in 200 μL FACS buffer. Data was acquired using a LSRFortessa flow cytometer (BD Biosciences) and data was analyzed using FlowJo version 10.0 (FlowJo LLC). Gating strategy is illustrated at S7 Fig.

### ROS and NO assays

For ROS assays, 5x10^5^ PMNs purified as described above were incubated for 16 hours with supernatants from HFF cells (uninfected or CL-14 or CL Brener-infected, collected at 4 dpi) in 8-chamber microscopy slides pre-treated with poly-lysine (Sigma Aldrich). After washes, cells were stimulated with 200 ng/mL LPS from *Escherichia coli* O111:B4 (Invivogen) for 1 hour. In the final 30 minutes of incubation, 5 μM DCF-DA (ABCam) was added. Cells were then immediately taken to a fluorescence microscope, and the fluorescence intensity in the acquired images were analyzed using ImageJ. For NO assays, 2x10^6^ PMNs were incubated with HFF cells supernatants for 16 hours and, after washes, were stimulated with 200 ng/mL LPS. The supernatant was then collected, and NO_2_ (one of the main breakdown products of NO) was quantified using the Griess method.

### Neutrophil migration assays

Cell migration assays were performed using Boyden chamber plates with 5 μm pores (ChemoTx, Neuro Probe). 29 μL of supernatants from HFF cells (uninfect or infected with *T*. *cruzi* CL-14 or CL Brener, at 4 dpi), or RPMI (negative control) or 500 ng/mL CCL2 (positive control) were added to the lower chamber, and 200.000 PMN cells were added to the upper chamber. The plates were then incubated at 37 ºC for 1.5 hour and the cells at the lower chamber were counted using a hemocytometer.

### Quantification of cytokines and chemokines in the sera from patients

Patients with well-defined clinical forms of Chagas disease, as well as non-Chagas individuals were enrolled in this cross-sectional study, which has the approval of the Ethical Committee from Federal University of Minas Gerais (COEP-UFMG–ETIC006/05). Patients were from Chagas disease endemic areas within Minas Gerais, Brazil, and have been evaluated at the outpatient clinic of the Federal University of Minas Gerais, under the responsibility of one of us (MCPN). Treatment and clinical care were offered to all volunteers, despite their enrollment in this research project. Volunteer patients were carefully selected to be unequivocally within the indeterminate and cardiac clinical forms of Chagas disease. Patients from asymptomatic clinical form (Indeterminate Chagas patients–Ind-Ch) had positive serology, lack of clinical manifestations or alterations upon all clinical, radiological and echocardiographic examination. Cardiac Chagas patients (Card-Ch) displayed positive serology, right and/or left ventricular dilation, global left ventricular dysfunction, alterations in the cardiac electric impulse generation and conduction upon electrocardiogram, chest x-rays and echocardiography. Individuals who displayed negative specific serological tests for Chagas disease, from the same geographical region, were included as non-infected group (control). Any other chronic inflammatory diseases, diabetes, heart/circulatory illnesses or bacterial infections were used as exclusion criteria. All individuals signed a written informed consent. Pregnant women and children were not enrolled in the study. Plasma from all individuals were analyzed to determine the production of IL-1β, CCL2, IL-8, and CSF3 using the Bio-Plex Pro Human Cytokine Standard 27-plex (Biorad, Hercules, CA, USA). Assay was performed following the manufacture’s recommendations. Comparisons between different groups were performed using Tukey-Krammer test. Differences that returned *p* values of less than or equal to 0.05 were considered statistically significant.

## Supporting information

S1 Fig Hierarchical clustering of RNA-Seq data. Hierarchical clusterization of Euclidean distance represented by a heatmap. The distance between the samples were represented in a blue-white scale.
(TIF)

S2 Fig Hierarchical clustering of differentially expressed genes identified exclusively during infection with CL Brener or during infection with both strains. Expression values of protein-coding genes in response to the infection with *T*. *cruzi* CL Brener or CL-14 were represented by a heatmap and the hierarchical unsupervised clusterization.
(TIFF)

S3 Fig Gene signatures of CL Brener and CL-14 infection. Expression values of protein-coding genes exclusively altered in response to the infection with *T*. *cruzi* CL Brener or CL-14, represented by a heatmap and the hierarchical unsupervised clusterization, revealed a subset of a gene signature associated with the infection with each strain.
(TIFF)

S4 Fig Class distribution of lncRNAs. (TIF)

S5 Fig IL-1β production in PMA-treated THP-I macrophages in response to CL-14 and CL Brener. IL-1β production was measured in THP-I cells infected with *T*. *cruzi* CL-14 or CL Brener (10 parasites per cell). Supernatants were collected at 24, 72, 120 and 168 hpi and IL-1β was quantified by ELISA. Data from three independent experiments (mean and s.e.m.).
(TIF)

S6 Fig LDH released after HFF-infection with *T*. *cruzi* clones CL-14 and CL Brener. HFF monolayers were infected with trypomastigotes from CL Brener and CL-14 and LDH released in the supernatants was quantified daily. * p < 0.05 (unpaired t-test). Data from two independent experiments (mean and s.e.m.).
(TIF)

S7 Fig Gating strategy for CD11b experiments. Images are representative of three independent experiments.
(TIF)

S8 Fig Enhanced expression of CD11b in human neutrophils upon incubation with IL-8 and G-CSF. Flow cytometry analysis of live neutrophils (CD16^+^CD66b^+^CD14^-^HLA-DR^-^) incubated for 16 hours with media only (A), G-CSF (500 pg/mL) (B), or IL-8 (1 ng/mL) (C). Percentage of cells expressing high levels of the activation marker CD11b (D) and mean fluorescent intensity (MFI) of CD11b (E). * p < 0.05 (one-way ANOVA with Tukey’s post-test). (A-C) Images are representative of three independent experiments. (D-E). Data from three independent experiments (mean and s.e.m.).
(TIF)

S9 Fig Uncroppred images of immunoblots presented in Fig 5D. (A) β-actin, (B) ASC, (C) Pro-IL-1β, (D) NLRP3, (E) Caspase-1, (F) Caspase-4, (G) Caspase-8, (H) Gasdermin D. Images are representative of three independent experiments.
(TIF)

S10 Fig Ficoll-purified PMNs. (A-B) Representative blood smears of a healthy donor before PMN purification (A) and after (B). (C-D) Quantification of leukocytes based on morphology before (C) and after PMN purification (D). (A-B) Images are representative of two healthy donors. (C) Data from two healthy donors (mean and s.e.m.). 200 cells were counted for each donor.
(TIF)

S1 Table Summary of samples and mapping information. The workbook contains one worksheet with all information related to mapping as samples ID, number of reads sequenced, mapped reads and their percentage related to human and *T*. *cruzi* genome.
(XLSX)

S2 Table Raw and normalized counts for all samples. The workbook contains three worksheets related to raw counts, Fragments per Million mapped reads (FPKM) and log2FPKM. All gene ids are related to *Human Genome* Assembly *GRCh37* obtained from Ensembl database.
(XLSX)

S3 Table Differentially expressed genes (DEGs) tables for all contrasts using DESeq2. The workbook comprises eleven worksheets related to legends, summary of DEGs tables, additional figures related to DEGs, 8 comparisons performed between infected and uninfected samples (comp1-4), between strains (comp5-6) and between time-points (comp7-8).
(XLS)

S4 Table Enriched canonical pathways tables using Ingenuity *Pathway* Analysis (*IPA*). The workbook comprises four worksheets related to enriched pathways analysis provided by IPA from all DEGs obtained from comparisons between infected and uninfected samples.
(XLSX)

S5 Table Differentially expressed long noncoding RNAs (DElncRNAs) tables for all contrasts during Y strain infection. The workbook comprises seven worksheets related to summary of DElncRNAs tables and 6 comparisons performed between HFF cells infected with Y strain (4, 6, 12, 24, 48 and 72 hpi) and uninfected samples previous analyzed by Li et al. (2016). For all comparisons we added the information if the gene was up-regulated (up), down-regulated (down) or no significant (no_sig) in our data related to HFF infection with CL Brener and CL-14. The annotation was obtained from Human Genome Assembly GRCh37 in Ensembl database.
(XLSX)
