# Supplementary figures and images for: Gene expression network analyses during infection with virulent and avirulent Trypanosoma cruzi strains unveil a role for fibroblasts in neutrophil recruitment and activation
Source: PLoS Pathog. 2020 Aug 18;16(8):e1008781. doi: 10.1371/journal.ppat.1008781 (PMC7508367; doi:10.1371/journal.ppat.1008781)

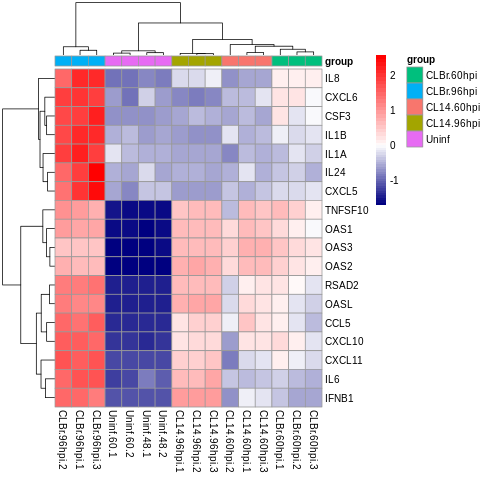

Supplement: S2 Fig — Expression values of protein-coding genes in response to the infection with T. cruzi CL Brener or CL-14 were represented by a heatmap and the hierarchical unsupervised clusterization. (TIFF) [file ppat.1008781.s002.tiff]

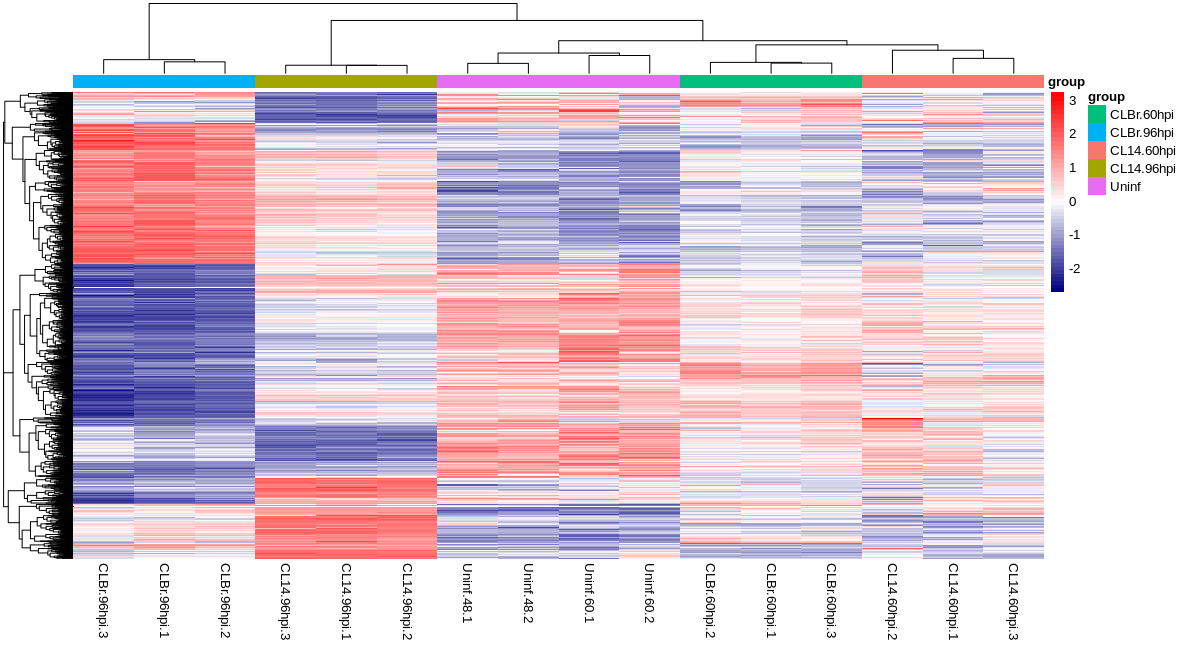

Supplement: S3 Fig — Expression values of protein-coding genes exclusively altered in response to the infection with T. cruzi CL Brener or CL-14, represented by a heatmap and the hierarchical unsupervised clusterization, revealed a subset of a gene signature associated with the infection with each strain. (TIFF) [file ppat.1008781.s003.tiff]

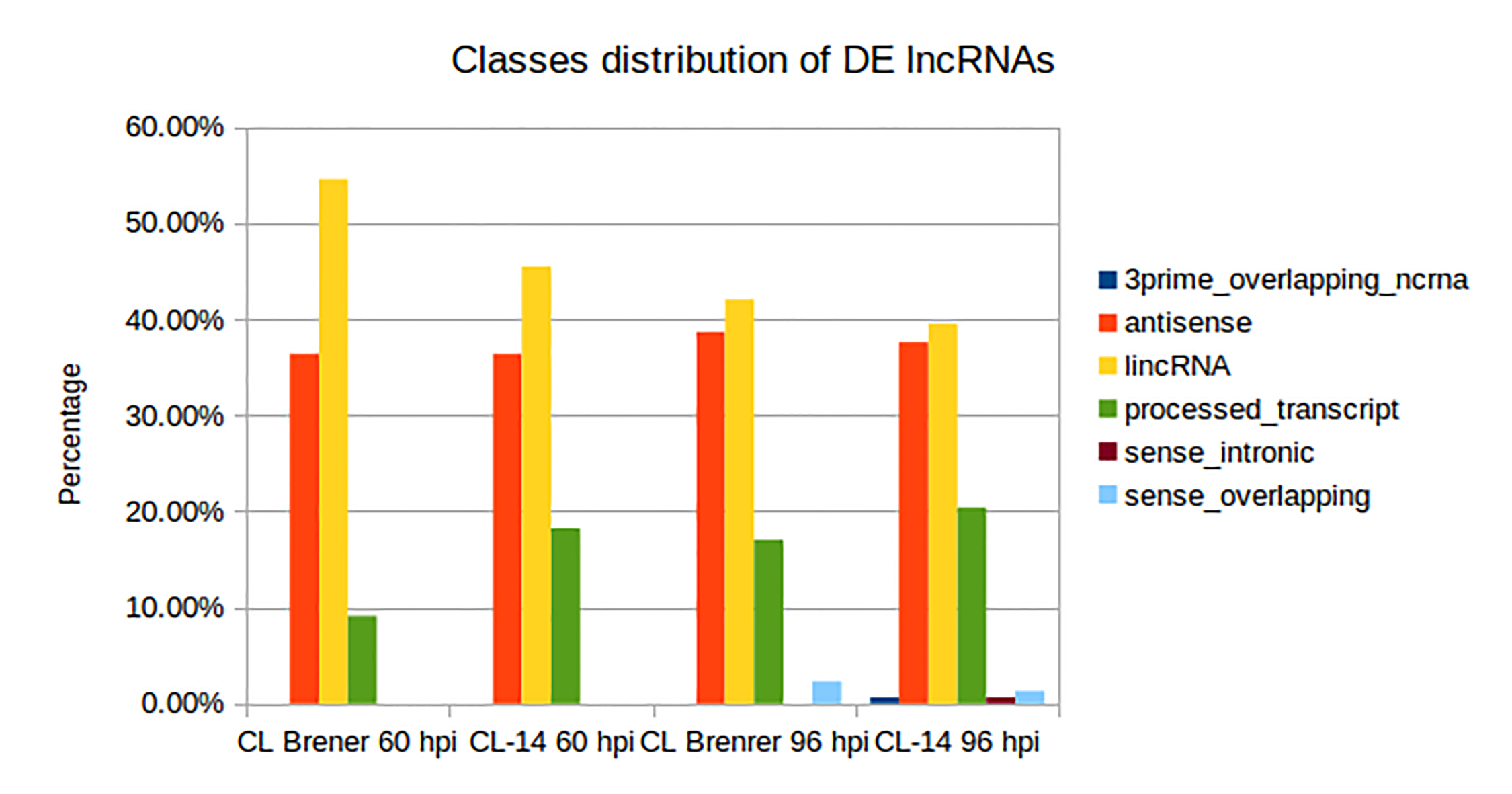

Supplement: S4 Fig — (TIF) [file ppat.1008781.s004.tif]

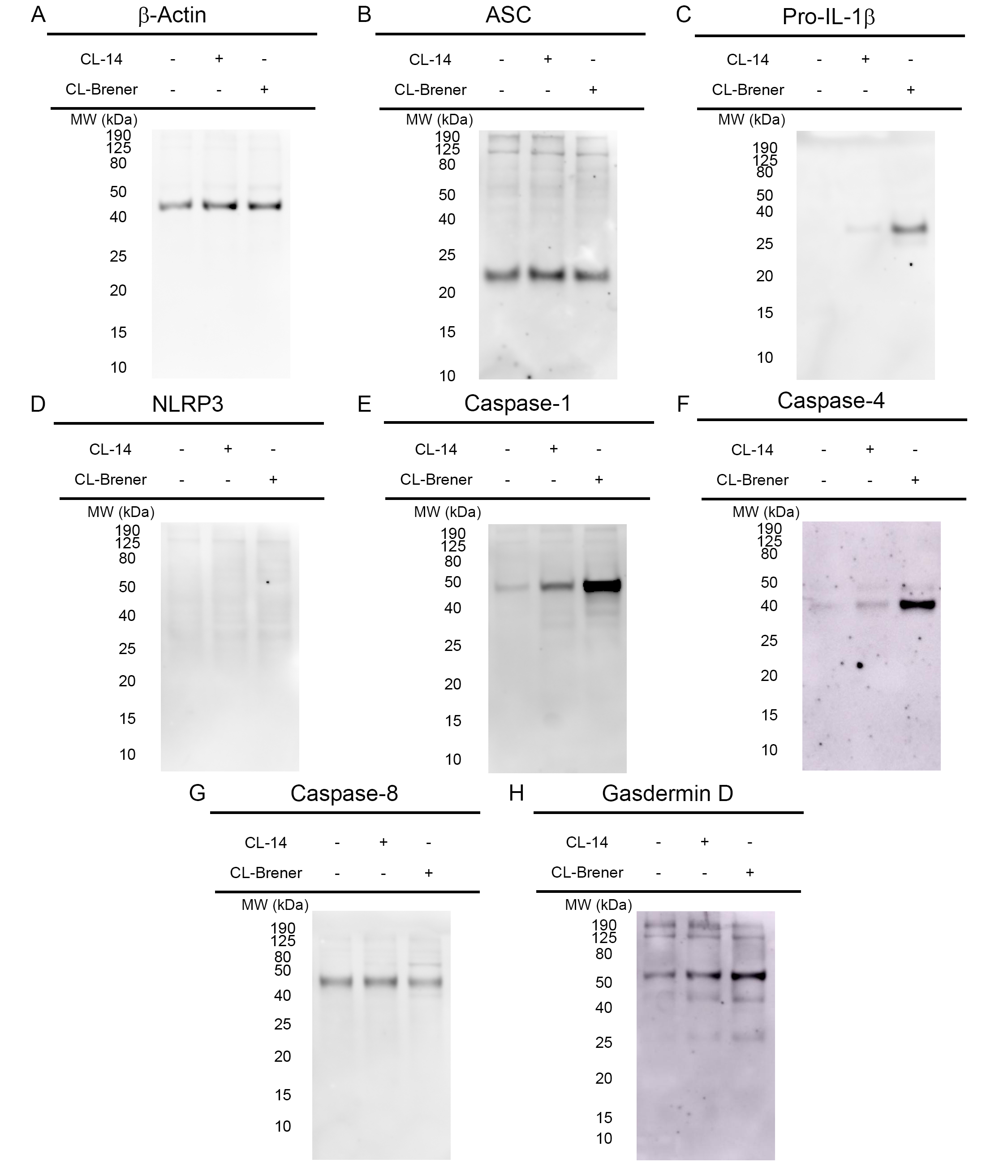

Supplement: S9 Fig — (A) β-actin, (B) ASC, (C) Pro-IL-1β, (D) NLRP3, (E) Caspase-1, (F) Caspase-4, (G) Caspase-8, (H) Gasdermin D. Images are representative of three independent experiments. (TIF) [file ppat.1008781.s009.tif]
